# RAP2.3 negatively regulates nitric oxide biosynthesis and related responses through a rheostat-like mechanism in Arabidopsis

**DOI:** 10.1093/jxb/eraa069

**Published:** 2020-02-13

**Authors:** José León, Álvaro Costa-Broseta, Mari Cruz Castillo

**Affiliations:** 1 Instituto de Biología Molecular y Celular de Plantas (Consejo Superior de Investigaciones Científicas–Universidad Politécnica de Valencia), Valencia, Spain; 2 University of Edinburgh, UK

**Keywords:** ABA signaling, *Arabidopsis thaliana*, jasmonate signaling, nitric oxide, oxidative stress, RAP2.3 transcription factor

## Abstract

Nitric oxide (NO) is sensed through a mechanism involving the degradation of group-VII ERF transcription factors (ERFVIIs) that is mediated by the N-degron pathway. However, the mechanisms regulating NO homeostasis and downstream responses remain mostly unknown. To explore the role of ERFVIIs in regulating NO production and signaling, genome-wide transcriptome analyses were performed on single and multiple *erfvii* mutants of Arabidopsis following exposure to NO. Transgenic plants overexpressing degradable or non-degradable versions of RAP2.3, one of the five ERFVIIs, were also examined. Enhanced *RAP2.3* expression attenuated the changes in the transcriptome upon exposure to NO, and thereby acted as a brake for NO-triggered responses that included the activation of jasmonate and ABA signaling. The expression of non-degradable RAP2.3 attenuated NO biosynthesis in shoots but not in roots, and released the NO-triggered inhibition of hypocotyl and root elongation. In the guard cells of stomata, the control of NO accumulation depended on PRT6-triggered degradation of RAP2.3 more than on RAP2.3 levels. RAP2.3 therefore seemed to work as a molecular rheostat controlling NO homeostasis and signaling. Its function as a brake for NO signaling was released upon NO-triggered PRT6-mediated degradation, thus allowing the inhibition of growth, and the potentiation of jasmonate- and ABA-related signaling.

## Teaser

RAP2.3 mediates NO sensing and promotes NO biosynthesis, but represses NO-triggered responses through a rheostat-like mechanism that depends on its degradation through the N-degron pathway.

## Introduction

Nitric oxide (NO) is endogenously produced in plants through multiple alternative pathways that include chemically driven processes and enzyme-based mechanisms, the latter being either reductive or oxidative (Astier *et al.*, 2017). Among the reductive pathways, the most relevant and probably the best studied involves the catalysis of nitrate reductases that convert nitrate to nitrite, and subsequently to NO (Gupta *et al.*, 2011). NO exerts a wide array of developmental regulatory functions in plants that span the complete life cycle, including seed dormancy and germination, skotomorphogenic and photomorphogenic vegetative development, flowering, fruiting, and senescence (Beligni and Lamattina, 2000; He *et al.*, 2004; Tsai *et al.*, 2007; Manjunatha *et al.*, 2010; Lozano-Juste and León, 2011; Arc *et al.*, 2013; Liu and Guo, 2013; Du *et al.*, 2014). In addition, NO also regulates plant defense responses against biotic and abiotic stresses (Mur *et al.*, 2006; Siddiqui *et al.*, 2011; Arasimowicz-Jelonek and Floryszak-Wieczorek, 2014; Fancy *et al.*, 2017), and against oxidative stress (Beligni and Lamattina, 1999; Mazid *et al.*, 2011; Thomas, 2015). Although the production of NO and its impact on plant physiology have been studied extensively, far less is known about the mechanisms by which NO is sensed and the downstream signaling pathways. In the absence of a guanylate cyclase functioning as a NO receptor, as is the case in mammals, plants seem to sense NO mostly through chemical interactions with co-factor metals or with sensitive amino acid residues of proteins that often undergo NO-triggered post-translational modifications, such as S-nitrosylation and nitration of cysteine and tyrosine, respectively (Astier and Lindermayr, 2012). A NO sensor mechanism involving the NO- and O_2_-dependent oxidation of transcription factors of the group-VII ethylene responsive factor family (ERFVIIs) has been reported in Arabidopsis (Gibbs *et al.*, 2014*b*). The oxidation of cysteine 2 (C2) of ERFVIIs is catalysed by plant cysteine oxidases that do not seem to require NO (White *et al.*, 2018; Puerta *et al.*, 2019). Therefore, it remains unclear as to what role NO has in the C2 oxidation-dependent control of ERFVII stability through regulation of its proteolysis. This proteolysis involves the proteasomal degradation through the N-degron pathway (Varshavsky, 2019), and requires the polyubiquitilation of the oxidized factors by an E3 ubiquitin ligase, named PRT6 in Arabidopsis (Gibbs *et al.*, 2014*a*; Dissmeyer *et al.*, 2018). The ERFVIIs comprise three constitutively expressed transcription factors (RAP2.2, RAP2.3/EBP, and RAP2.12) and two hypoxia-inducible factors (HRE1 and HRE2) that all contain a cysteine residue immediately after the initial methionine, which have been demonstrated to be substrates of the PRT6 N-degron pathway (Gibbs *et al.*, 2015).

RAP2.3/EBP was originally identified as a suppressor of Bax-induced cell death by functional screening in yeast, and its activity in suppressing cell death has subsequently been confirmed together with its capacity to induce resistance to hydrogen peroxide and heat stress (Ogawa *et al.*, 2005). RAP2.3 also seems to regulate defense against pathogens. probably through interactions with the acyl-CoA binding proteins ACBP2 and 4 (Li *et al.*, 2008), and also with bZIP transcription factors (Büttner and Singh, 1997). In addition, RAP2.3 and the other members of ERFVII group have been extensively characterized as key regulators in the expression of hypoxia-responsive genes related to anaerobic metabolism that are involved in different abiotic stresses (Bui *et al.*, 2015; Papdi *et al.*, 2015; Gasch *et al.*, 2016). It has recently been reported that NO controls RAP2-3 stability and that this process is required for the ethylene-mediated pre-adaptation of plants to hypoxia stress (Hartman *et al.*, 2019). RAP2.3 also has functions related to the regulation of development. It has been identified as interacting with the GIBBERELLIN INSENSITIVE (GAI) DELLA protein, thereby impairing its activity on the promoters of target genes that control differential growth during apical hook development (Marín-de la Rosa *et al.*, 2014; Abbas *et al.*, 2015).

Despite the accumulation of information on the functional interactions between NO and RAP2.3 in controlling plant responses, there has as yet been no analysis of the impact of the functioning of RAP2.3 on NO homeostasis and on the NO-responsive transcriptome. Here, we examined the way RAP2.3 can modulate NO homeostasis and signaling. Our findings indicate that RAP2.3 controls NO homeostasis through a rheostat-like mechanism, and that it acts mostly as a repressor of NO-triggered responses both at the physiological and molecular levels.

## Materials and methods

### Plant material, growth conditions, and NO treatment

Transgenic lines overexpressing MC-RAP2.3-HA and MA-RAP2.3-HA as well as the single *rap2.3*, double *rap2.3rap2.12*, and quintuple *erfvii* mutants were all in the wild-type *Arabidopsis thaliana* Col-0 background as previously reported (Gibbs *et al.*, 2014*b*). TPT_RAP2.3 and TPT_RAP2.12 transgenic lines, conditionally expressing *RAP2.3* or *RAP2.12* under the control of a promoter inducible by β-estradiol (Coego *et al.*, 2014), were used as described in the MIAME section of Supplementary Table S2 at *JXB* online, with 10 μM β-estradiol treatment to induce transgene expression. Seeds were sown in moistened soil and grown under long-day conditions of 16/8 h light/dark at 22/20 °C with 150 µE m^−2^ s^−1^ (provided by cool-white fluorescent lamps) and 60 % relative humidity. In other experiments, surface-sterilized seeds were sown after 4 d of stratification at 4 °C under darkness and grown in agar-supplemented Murashige and Skoog (MS) medium supplemented with 1% (w/v) sucrose. A pulse of NO was applied by incubating plants for 5 min in a tightly sealed transparent box injected with pure NO gas (Linde AG, Germany) to a concentration of 300 ppm.

### Assays for NO-triggered inhibition of hypocotyl elongation

Surface-sterilized seeds were sown in MS-MES media supplemented with 1% sucrose, stratified for 4 d at 4 °C under darkness, and then germination was activated by exposure to light for 6 h. The plants were then incubated in sealed boxes in darkness with air supplemented to 300 ppm NO for an additional 4 d. Control seedlings were incubated under the same conditions in air with no supplemented NO. Hypocotyl lengths were measured for all seedlings using ImageJ. The experiments were repeated three times with at least 20 individuals per genotype–treatment combination. HA-tagged proteins were separated by denaturing 12% PAGE, blotted to nitrocellulose membranes, analysed by western blotting with anti-HA-HRP (1:2000 dilution), and detected using a Select ECL system (GE).

### NO detection by fluorescence

The endogenous levels of NO in shoots and roots were determined by staining with 10 µM 4-amino-5-methylamino-2’,7’-difluorofluorescein diacetate (DAF-FM DA) as described by Guo *et al.* (2003), with some modifications. Fluorescence was detected by confocal microscopy using a LSM 780 (Zeiss) or fluorescence microscopy with a MacroFluo MZZ16F system (Leica), using unchanged parameters for every measurement. NO specificity of the staining was achieved by pre-incubation of samples for 2 h with 250 µM of the NO scavenger 2-(4-carboxyphenyl)-4,4,5,5-tetramethylimidazoline-1-oxyl-3-oxide (cPTIO). Plants treated with 0.1 mM salicylic acid (SA) for 2 h were used as positive controls for induced NO accumulation. Quantification of the fluorescence was achieved by counting green pixels using ImageJ on images from at least four plants per genotype–treatment combination.

### RNA isolation, and RT-qPCR and transcriptomic analyses

TPT_RAP2.3 and TPT_RAP2.12 plants were grown *in vitro* in MS supplemented with 10 μM β-estradiol (EST)under 16/8 h light/dark conditions for 10 d and were then exposed to a pulse of 300 ppm NO for 5 min as described above. Control plants were grown in MS medium without EST. After 1 h, total RNA was extracted from samples of ~10 plants and purified using a Nucleospin RNA Plant kit (Macherey-Nagel), reverse-transcribed with M-MuLV Reverse transcriptase (RNase H minus) and oligodT, and the resulting cDNAs were quantified by real-time quantitative PCR (RT qPCR) using 7500 Fast Real-Time thermocyclers (Applied Biosystems) by using specific primer pairs (Supplementary Table S1). For microarray analyses, seedlings at 1 h after the NO pulse were frozen in liquid nitrogen and the total RNAs were extracted with Trizol and purified with a RNeasy kit (Qiagen). RNAs (three independent biological replicates per genotype, each consisting of ~10 plants) were checked for their integrity and purity by nanocapillary electrophoresis using an Agilent Bioanalyzer 2100. The transcriptomes were analysed using the Arabidopsis Agilent microarray platform. Labeling, hybridization protocols, and statistical analyses are included in a detailed MIAME rules-based description of the microarray experiments in Supplementary Table S2. The *Actin2* (*ACT2*) gene was used as the internal reference.

### Statistical analyses

Differential gene transcript levels and hypocotyl lengths were analysed using Student’s *t*-test. Linear Model Methods (LiMMA) were used for determining differentially expressed genes in microarray-based analyses. To control the false-discovery rate, *P*-values were corrected using the method of Benjamini and Hochberg (1995). Criteria for selection of genes were fold value >1.5 and false-discovery rate ≤0.05.

### *In silico* analyses of gene ontology and promoter motifs

Gene Ontology (GO) enrichment of functional categories in gene lists was performed using the Gene Ontology Consortium tools (http://www.geneontology.org/). A search for the RAP2.3 binding motif MGCCGYM in the promoter sequences of the Arabidopsis genome was performed using the Patmatch tool in the TAIR10 Loci Upstream Sequences–1000 bp (DNA) database.

## Results

### A rheostat-like mechanism based on RAP2.3 degradation controls endogenous NO content

**Fig. 1.**
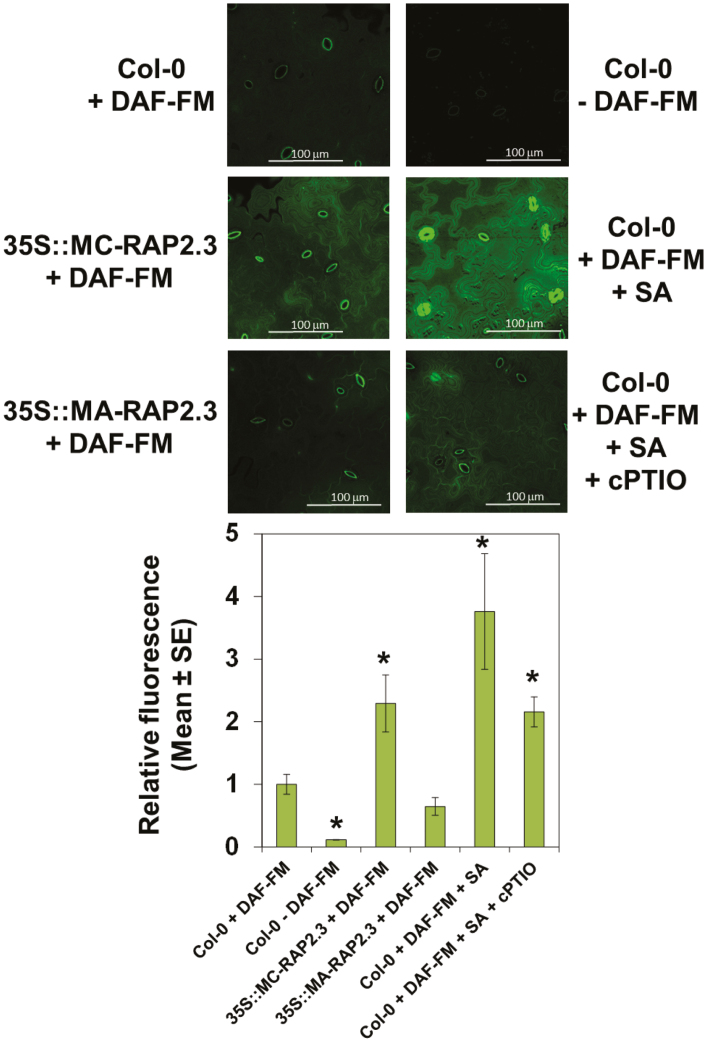
Distribution of NO in cotyledons of transgenic Arabidopsis plants overexpressing either degradable MC-RAP2.3 or the non-degradable form MA-RAP2.3, compared with the wild-type Col-0. Cotyledons were treated (+) or untreated (–) with 10 µM DAF-FM diacetate, 250 µM of the NO scavenger cPTIO, and 100 µM of the NO inducer salicylic acid (SA) as indicated. Images are representative of 3–5 replicate experiments. Fluorescence was detected by confocal microscopy with *Z*-stacks equivalents and is expressed relative to the value for Col-0+DAF-FM, which was set as 1. Data are means of *n*=4 replicates. Significant differences compared to Col-0+DAF-FM were determined using Student’s *t*-test: *P<0.05..

**Fig. 2.**
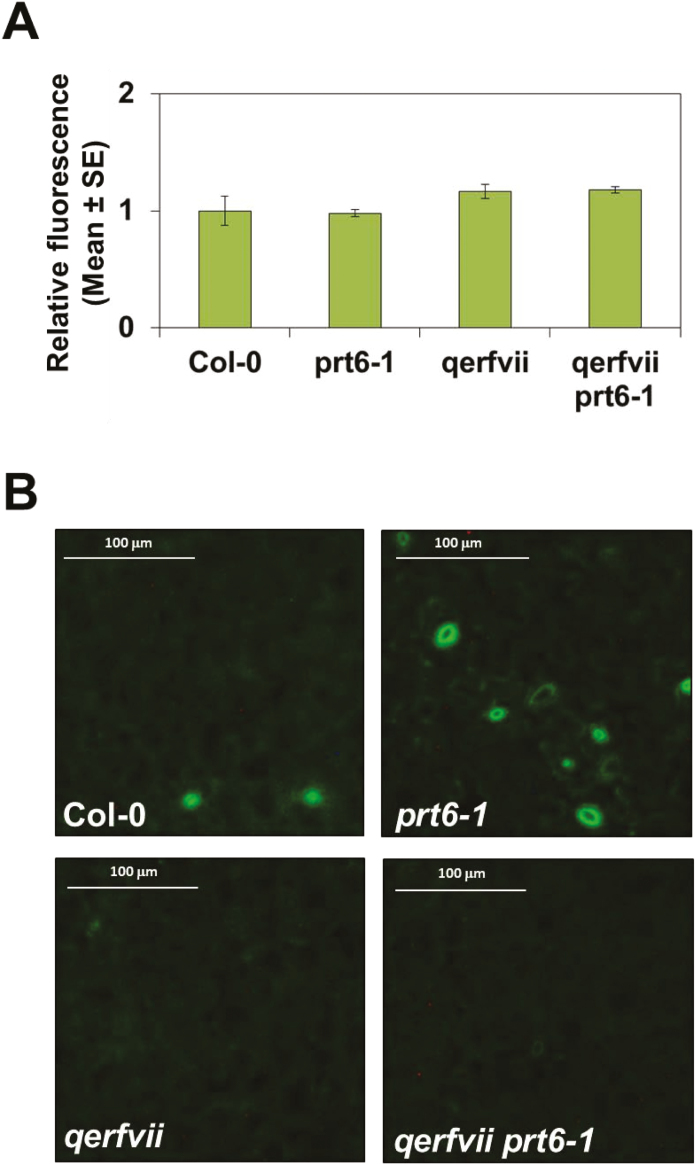
Distribution of NO in cotyledons of Arabidopsis N-degron pathway mutants and the Col-0 wild-type. *qerfvii* plants are quintuple mutants with loss of function of all ERFVII transcription factors. Cotyledons were treated with 10 µM DAF-FM DA. (A) Fluorescence was detected by confocal microscopy with *Z*-stacks equivalents and is expressed relative to the value for Col-0, which was set as 1. Data are means of *n*=3 replicates. (B) Images are representative of three replicate experiments.

RAP2.3 is one of the members of the group-VII ERF transcription factors previously reported to be involved in NO sensing (Gibbs *et al.*, 2014*b*). As a substrate of the PRT6 branch of the N-degron pathway of proteolytic degradation (Gibbs *et al.*, 2011; Licausi *et al.*, 2011), the stability of the RAP2.3 protein depends on its N-terminal sequence, in such a way that a wild-type version containing the MC N-terminal motif is degraded by the proteasome following polyubiquitylation, whereas a mutated MC2A version is resistant to proteasome-mediated degradation. By using plants overexpressing either MC-RAP2.3 or MA-RAP2.3 (Gibbs *et al.*, 2014*b*), we examined the effects of RAP2.3 on the endogenous NO content. After staining with the NO-specific fluorophore DAF-FM, plant cotyledons overexpressing the degradable MC-RAP2.3 version showed a 2.3-fold increase in NO-associated fluorescence compared to wild-type plants (Fig. 1); however, the levels in the roots of *MC-RAP2.3*-overexpressing (-OX) plants were not significantly different than those of the wild-type (Supplementary Fig. S1A). Higher levels of fluorescence were detected in plants treated with SA (3.8-fold; Fig. 1), which has been reported to be a strong inducer of NO production both in roots and shoots (Zottini *et al.*, 2007). Fluorescence was confirmed to be associated with the endogenous content of NO, as samples treated with both SA and the NO-specific scavenger cPTIO showed lower fluorescence levels than plants treated with SA alone (Fig. 1). The enhanced NO content in *MC-RAP2.3*-OX plants was not directly associated with the protein expression levels, as overexpression of the non-degradable MA-RAP2.3 version led to cotyledon fluorescence that was not significantly different to that of the wild-type. These results pointed to a requirement for degradation of RAP2.3 in order to release a brake on NO biosynthesis. Since RAP2.3 degradation by the proteasome requires prior polyubiquitylation mediated by the E3 ubiquitin ligase PRT6, we examined whether the NO content in cotyledons was altered in *prt6-1* mutants. The overall fluorescence associated with NO in *prt6-1* plants was not significantly different from that in the wild-type plants, either in the cotyledons (Fig. 2A) or in the roots (Supplementary Fig. S1B), thus suggesting that stabilization of RAP2.3 did not lead to an increase in NO content. However, increases in NO levels were detected in the guard cells of stomata (Fig. 2B), in agreement with a previous report (Gibbs *et al.*, 2014*b*). Interestingly, the quintuple-mutant with loss of function of all ERFVII transcription factors (*qerfvii*), which contained NO levels similar to the wild-type both in shoots (Fig. 2A) and roots (Supplementary Fig. S1B), displayed almost no fluorescence in the stomata (Fig. 2B). A similar pattern of endogenous NO content was also detected in the combined *qerfvii prt6-1* mutant (Fig. 2, Supplementary S1B). These results suggested that NO synthesis and accumulation in stomatal guard cells were regulated by RAP2.3 in a different manner to that in other leaf cells. NO content in the stomata appeared to depend on the levels of RAP2.3 whereas in other leaf cells it appeared to be regulated by degradation through the PRT6 N-degron pathway. These findings pointed to a potential role for RAP2.3 working as a molecular rheostat, in such a way that the synthesis of NO in non-stomatal cells would be regulated not by the actual levels of the protein but through the capacity of RAP2.3 to be degraded through the N-degron pathway.

### RAP2.3 modulates NO sensing in shoots and roots

**Fig. 3.**
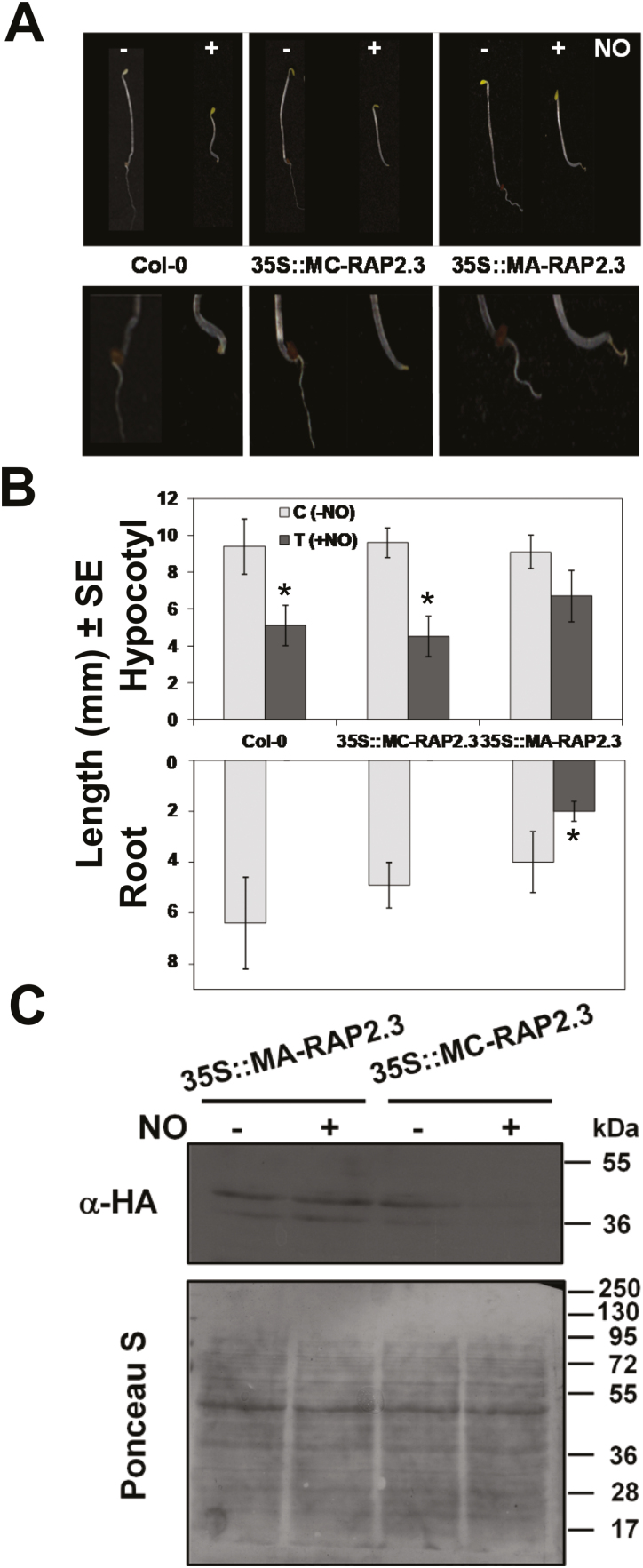
Overexpression of non-degradable MA-RAP2.3 confers hyposensitivity to NO in Arabidopsis. (A) Hypocotyls (upper panels) and roots (lower panels) of etiolated seedlings either with (+) or without (–) NO treatment (300 ppm) for transgenic plants overexpressing either MA-RAP2.3 or the degradable form MC-RAP2.3, compared with the wild-type Col-0. (B) Lengths of hypocotyls and primary roots of the transgenic plants compared with Col-0. ImageJ was used to measure the lengths for at least 20 seedlings. Significant differences between control (C, –NO) and treated (T, +NO) plants were determined using Student’s *t*-test: *P<0.05. (C) Levels of RAP2-3-HA in protein extracts from etiolated seedlings with NO (+) or without (–) NO treatment, as determined by western blotting with anti-HA antibodies. Total protein staining with Ponceau S is shown as the loading control. The position of molecular weight markers is shown at the right.

Consistent with the central role exerted by the E3 ubiquitin ligase PRT6 in NO sensing through its branch of the N-degron pathway (Gibbs *et al.*, 2014*b*, 2014*b*), we have previously reported that the NO-triggered inhibition of hypocotyl elongation is impaired in the *prt6* mutant (Gibbs *et al.*, 2014*b*). Thus, the stabilization of ERFVIIs seems to be a key factor for the sensitivity of plant hypocotyls to NO. By using plants overexpressing either the wild-type MC-version or the mutated MA-version of RAP2.3, we used etiolated hypocotyl growth assays to examine whether enhanced levels of RAP2.3 altered the sensitivity to NO. We found that plants overexpressing the MC-RAP2.3 version were as sensitive to NO-triggered inhibition of hypocotyl elongation as the wild-type (Fig. 3A, B). In contrast, the overexpression of the non-degradable MA-RAP2.3 version almost fully released the NO-triggered inhibition of elongation. To check whether these differential phenotypes were related to the stability of the RAP2.3 version expressed, we took advantage of the C-terminal HA-tags of both proteins. Whereas MC-RAP2.3 was efficiently degraded by NO, the levels of MA-RAP2.3 remained high under NO treatment (Fig. 3C), thus correlating with the NO-sensitive and -resistant phenotypes observed. In addition to shortening of the hypocotyl, the response to NO was also characterized by a drastic inhibition of primary root growth in the wild-type plants (Fig. 3A, B). *MC-RAP2.3*-OX plants were completely inhibited in root growth whereas *MA-RAP2.3*-OX plants displayed significant (albeit still defective) root growth under the NO treatment. These results therefore suggested that stabilization of the RAP2.3 protein made shoots and roots less sensitive to NO.

### Genome-wide transcriptome analyses reveal RAP2.3 as a general negative regulator of NO-triggered responses

**Fig. 4.**
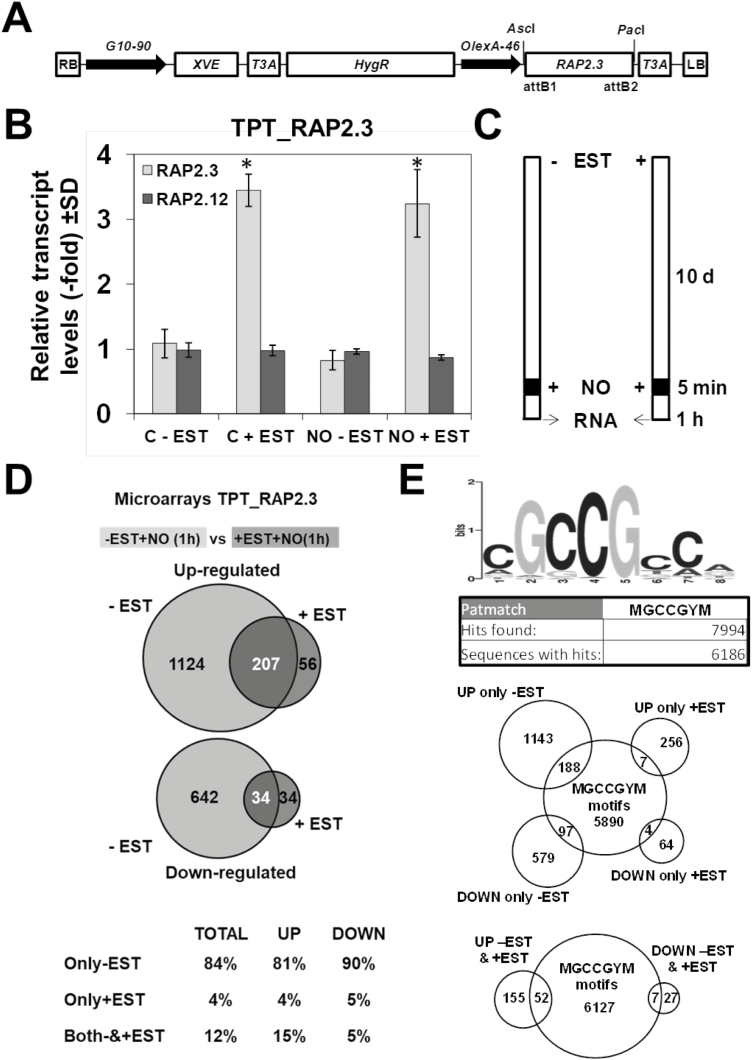
Transcriptome analysis of Arabidopsis *RAP2.3* transgenic lines inducible by β-estradiol. (A) Construct used to conditionally express *RAP2.3* under the control of a β-estradiol-inducible *XVE* factor that activates the *OlexA-46* promoter. (B) *RAP.3* and *RAP2.12* transcript levels in control (C) plants and plants treated with NO in the presence (+) or absence (–) of β-estradiol (EST), as determined by RT-qPCR with specific primers. The *Actin 2* (*ACT2*) gene was used to normalize the results, and transcript levels are expressed relative to the value in non-treated control (C) plants, which was set as 1. Data are means of three independent biological replicates. Significant differences compared with not treated control (C) plants were determined using Student’s *t*-test: *P<0.05. (C) Schematic diagram of the time-course of the experiment. (D) Venn diagrams showing differentially up- and down-regulated genes in conditionally expressing TPT-RAP2.3 transgenic lines with (+) or without (–) treatment with the transgene inducer EST. (E) The putative RAP2.3-binding element and *in silico* analysis of its presence in gene promoters across the whole Arabidopsis genome. The Venn diagrams represent the intersections between genes that were identified as differentially expressed in NO-treated plants and those carrying the binding motif in their promoters.

Inhibition of root and hypocotyl growth is only part of the NO-triggered responses in plants. We have previously reported that a pulse of NO triggers a transient but extensive metabolic reprogramming that includes enhanced levels of polyamines, lipid catabolism, and accumulation of phospholipids, chlorophyll breakdown, protein and nucleic acid turnover, and decreased and increased contents of starch and sugars, respectively (León *et al.*, 2016). To assess how NO triggers multiple molecular responses and the regulatory role exerted by RAP2.3 on those responses, we conducted comparative transcriptome analyses of NO-treated versus untreated plants in Arabidopsis TPT (TRANSPLANTA) transgenic lines (Coego *et al.*, 2014) that conditional express *RAP2.3* under a β-estradiol-inducible promoter (Fig. 4A). Upon treatment with β-estradiol (EST), these plants specifically expressed *RAP2.3*, as demonstrated by the large accumulation of its transcript and the absence of enhanced expression of the very closely related *RAP2.12* gene (Fig. 4B). Following the experimental scheme shown in Fig. 4C, EST-treated and untreated control plants were grown for 10 d under standard growing conditions, exposed to a 300-ppm pulse of NO for 5 min, and 1 h later samples were collected for RNA isolation and further transcriptome analyses. Normalized and filtered data (*P*-value corrected for FDR<0.05, and fold-change in absolute values >1.5) from all transcriptome analyses are summarized in Supplementary Table S2. The NO pulse triggered the rapid and differential regulation of 2097 genes, representing ~10% of the Arabidopsis genome (Fig. 4D), thus pointing to associated transcriptional regulation of the NO-induced responses. However, these responses at the transcriptome level were largely attenuated when NO-treated plants over-expressed *RAP2.3* upon activation by EST treatment. Of the genes regulated by NO, ~81% of those up-regulated and ~90% of those down-regulated in TPT_RAP2.3 plants were differentially expressed only in the absence of EST (Fig. 4D, Supplementary Table S2). The 1124 and 642 genes that were up- and down-regulated, respectively, by NO in plants in the absence of EST but not in those overexpressing *RAP2.3* represent potential NO targets that are negatively regulated by RAP2.3. By contrast, only 263 and 68 genes were up- and down-regulated, respectively, by NO regardless of the levels of *RAP2.3* expression (Fig. 4D). Of those, 56 and 34 genes were up- and down-regulated, respectively, by NO only when *RAP2.3* was overexpressed, thus suggesting that RAP2.3 might act also as a positive regulator of NO-triggered changes in this subset of genes. These results suggest that in addition to playing a role in controlling NO biosynthesis and sensing, RAP2.3 exerted a mostly negative effect on the NO-regulated transcriptome. An *in silico* screening of GCC-like boxes, defined previously as putative RAP2.3-binding motifs (Franco-Zorrilla *et al.*, 2014), was performed. Searching the 1000-bp promoter sequence upstream of the initiation codon of genes in the whole Arabidopsis genome for the (C/A)GCCG(C/T)(C/A) motif sequence resulted in 7994 hits corresponding to 6186 sequences (Fig. 4E, Supplementary Table S3). Among these putative RAP2.3-binding targets, 296 corresponded to genes that were differentially regulated in NO-treated plants of the TPT_RAP2.3 transgenic lines. We found 188 and 97 genes containing the consensus RAP2.3-binding motif that were up- or down-regulated, respectively, by NO only in TPT plants that were not treated with EST. By contrast, only seven and four genes containing the consensus motif were up- or down-regulated, respectively, by NO only in TPT plants that were treated with EST (Fig. 4E, Supplementary Table S3), thus suggesting that the number of targets negatively regulated by RAP2.3 in NO-triggered responses was far larger than that of positively targets. We also found a relatively large set of genes carrying putative RAP2.3-binding motifs, with 52 and seven that were up- or down-regulated, respectively, by NO in TPT_RAP2.3 plants independently of EST treatment (Fig. 4E, Supplementary Table S3), thus suggesting they were not truly targets of RAP2.3 regulation.

We also examined whether the repressing function on NO-triggered gene expression was specifically exerted by RAP2.3 or also by other members of the ERFVII group. Following a similar experimental scheme as described for RAP2.3 (Fig. 4A), we used three TPT lines conditionally expressing RAP2.12 (Coego *et al.*, 2014) and analysed the transcriptomes of NO-treated plants in the presence and absence of EST (Supplementary Table S2). The number of differentially expressed genes (DEGs) followed a similar pattern for NO-treated both TPT_RAP2.12 and TPT_RAP2.3 plants, although the number of DEGs that were up-regulated by NO in EST-treated TPT_RAP2.12 plants was higher than for TPT_RAP2.3 plants (Supplementary Fig. S2). These results suggested that RAP2.12 also exerted a repressive function on NO-triggered gene expression, although to a lesser extent than RAP2.3. In addition, the up- and down-regulation by NO of only 92 and eight genes, respectively, was repressed by both RAP2.3 and RAP2.12, thus suggesting these ERFVIIs may act mostly on different targets. Only 12–19% of the genes in the different subsets contained MGCCGYM sites in their promoter sequences (Supplementary Fig. S2) and hence represented potential primary targets of the repressive function of ERFVIIs.

### Gene-specific hormone signaling pathways that are NO-sensitive and independent of RAP2.3

The transcriptome of 241 genes that were responsive to NO and independent of RAP2.3 (Fig. 4D) was enriched in GO functional categories related to the biosynthesis, metabolism, and signaling of jasmonic acid (JA), as well as in categories related to responses to ethylene stimulus (Supplementary Table S2). The set of JA-related genes included some that code for biosynthetic enzymes such as LOX3, LOX4, AOC1, AOC3, OPR3, and OPCL1, and some that code for different components of JA signaling including the transcription factor MYC2 and the negative regulators JAZ2, 5, 6, 7, 9, 10, CYP94b1, and CYP94c1, all of which were strongly up-regulated by at least 6-fold up to more than 50-fold. Similarly, several ethylene response-related genes such as those coding for the ethylene response (ERF) transcription factors RRTF1, ERF13, ERF-2, ERF6, ERF-1, CEJ1/DEAR1, and RAP2.9/DEAR5 were also included in the group of 241 genes. Most of them were also connected to JA signaling and responses to oxidative stress. Our previous results indicated that mutations in both positive and negative regulators of JA signaling do not significantly alter the NO-triggered inhibition of hypocotyl elongation (Castillo *et al.*, 2018), thus suggesting these regulatory components of JA signaling are not involved in NO-triggered responses. Our current GO analysis found that ABA-related processes were also over-represented among up-regulated genes. This subset included genes coding for several dehydrins, such as ERD10, ERD12/AOC1, ERD15 or the E3 ubiquitin ligases PUB23 and PUB24, as well as genes coding for transcription factors related to water stress, such as MYBR1, MYC2, MBF1C, and DREB2b (Supplementary Table S2).

### Gene-specific hormone signaling pathways that are NO-sensitive and are positively regulated by RAP2.3

In addition to the NO-responsive genes that were independent of RAP2.3, we found two other sets that showed patterns of regulation by RAP2.3. The smaller set comprised 90 genes that required the overexpression of *RAP2.3* to be up- or down-regulated by NO (Fig. 4D). Notably, within this set the gene encoding the negative regulator of cytokinin ARR22 was strongly downregulated, as well as the genes encoding the auxin-responsive proteins SAUR29, 65, and 19, and the auxin metabolic IAA carboxylmethyltransferase 1 (IAMT1) and IAA-amido synthetase GH3.9 (Supplementary Table S2). By contrast, the gene encoding the transcription factor MYB77 that enhances auxin signaling was up-regulated by NO only in *RAP2.3*-OX plants. Taken together, these results suggested that NO may regulate the responses to auxins and cytokinins through RAP2.3-mediated processes. However, only seven and four genes among those up- and down-regulated, respectively, by NO in EST-treated plants contained the (C/A)GCCG(C/T)(C/A) motif in their promoters and, of the genes detailed above, only *SAUR65* was related to auxin or cytokinin signaling (Supplementary Table S3). We examined the sensitivity of wild-type, mutant, and transgenic plants with altered ERFVII function in root elongation assays in the presence of cytokinin or auxin. No significant alterations were found in root elongation in *rap2.3* and *rap2.3 rap2.12* mutant plants or in transgenic plants overexpressing *MC-RAP2.3* or *MA-RAP2.3* (Supplementary Fig. S3). Only the loss of function of all five ERFVIIs in the quintuple *qerfvii* mutant led to a slight but significant enhanced sensitivity to zeatin and indolebutyric acid, thus suggesting the altered expression of a single *ERFVII* gene was not enough to modify the response to these hormones. The phosphate transporters PHT1;4 and PHT2;1 were also down-regulated by NO in *RAP2.3*-OX plants (Supplementary Table S2). Interestingly, low phosphate and hypoxia have been reported to induce the alternative oxidase-mediated regulation of NO production and signaling in plants (Kumari *et al.*, 2019), thus representing a potential link between phosphate, NO, and ERFVII-regulated responses related to hypoxia. With regards to up-regulated genes in this set, we found that those encoding the transcription factors RAV1, BBX20/BZS1, and WRKY28 were only induced by NO in plants that overexpressed RAP2.3 (Supplementary Table S2), and these are involved in regulating ABA-related stress signaling, brassinosteroid and strigolactone signaling, and SA biosynthesis, respectively (van Verk *et al.*, 2011; Feng *et al.*, 2014; Wei *et al.*, 2016). We have previously found that NO sensing in hypocotyls requires ethylene, strigolactone, salicylate, and brassinosteroid signaling (Castillo *et al.*, 2018); however, again none of those genes contained RAP2.3-related motifs in their promoters (Supplementary Table S3), hence suggesting that the regulatory effects exerted by RAP2.3 would not be direct on these targets.

### Gene-specific hormone signaling pathways that are NO-sensitive and are repressed by RAP2.3

**Fig. 5.**
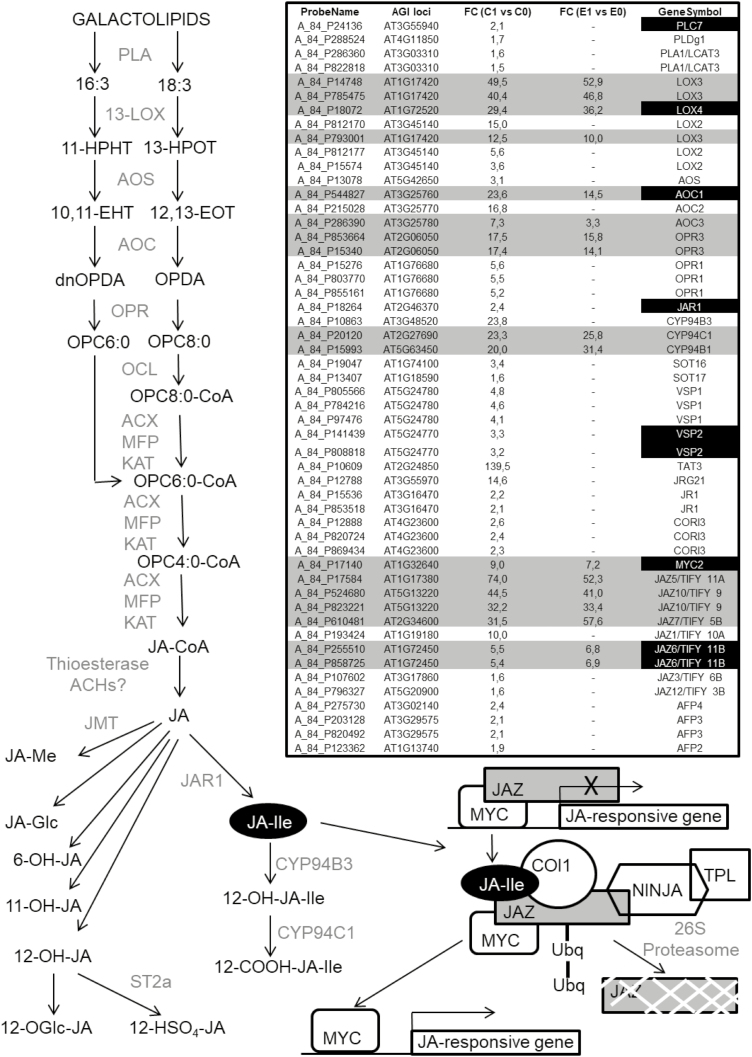
Effects of NO and RAP2.3 on the expression of jasmonate biosynthesis and signaling genes in Arabidopsis *RAP2.3* transgenic lines inducible by β-estradiol (EST). The diagram shows the jasmonate (JA) biosynthesis and signaling pathway. The table shows the fold-change (FC) values for the different transcripts in comparisons between plants at 1 h and 0 h after exposure to NO without EST treatment (C1 versus C0), and the same comparison between plants treated with EST (E1 versus E0). NO-regulated genes that were not significantly affected by *RAP2.3* overexpression are highlighted in grey. Genes highlighted in black contained the putative RAP2.3-binding sites in their promoters.

**Fig. 6.**
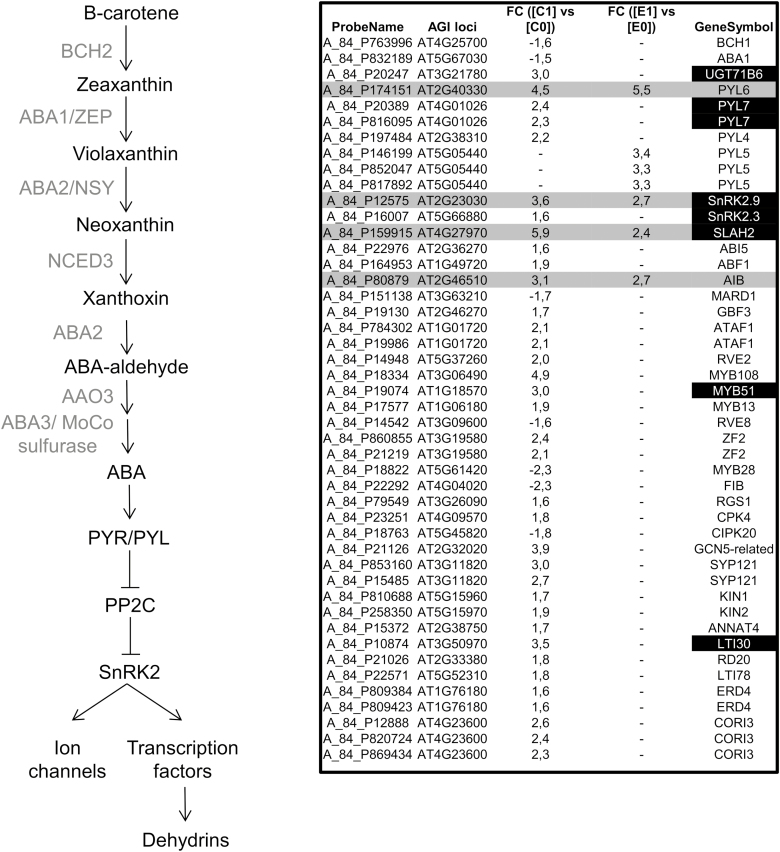
Effects of NO and RAP2.3 on the expression of ABA biosynthesis and signaling genes in Arabidopsis *RAP2.3* transgenic lines inducible by β-estradiol (EST). The diagram shows the biosynthesis and signaling ABA pathway. The table shows the fold-change (FC) values for the different transcripts in comparisons between plants at 1 h and 0 h after exposure to NO without EST treatment (C1 versus C0), and the same comparison between plants treated with EST (E1 versus E0). NO-regulated genes that were not significantly affected by *RAP2.3* overexpression are highlighted in grey. Genes highlighted in black contained the putative RAP2.3-binding sites in their promoters.

The second set of genes, and by far the largest group, comprised those that were up- or down-regulated in NO-treated plants only when *RAP2.3* was not overexpressed, thus suggesting that RAP2.3 acted as a negative modulator of the NO-triggered regulation for these genes. The GO analysis of this set indicated over-representation of functional categories related to responses to chitin, jasmonates, ABA, temperature, light, and salicylate stimuli (Supplementary Table S2). JA-related genes coding for biosynthetic and metabolic enzymes (*PLC7*, *PLDg1*, *PLA1/LCAT3*, *LOX2*, *AOS/CYP74A*, *AOC2*, *OPR1*, *JAR1*, *SOT16*, *CYP94C3*, *CYP94B1*), for repressors and co-repressors of signaling (*JAZ1*, *JAZ3*, *JAZ12*, and the NINJA-like *AFP2*, *AFP3*, *AFP4*), and for JA-responsive markers (*VSP1*, *VSP2*, *TAT3*, *JRG21*, *JR1*, *CORI3*) were all up-regulated by NO only when *RAP2.3* was not overexpressed (Fig. 5), thus suggesting that there was a complete JA biosynthesis and signaling pathway induced by NO and repressed by RAP2.3. Remarkably, only jasmonate biosynthesis genes *PLC7*, *LOX4*, and *JAR1* contained (C/A)GCCG(C/T)(C/A) motifs in their promoters (Supplementary Table S3); *JAR1* is a key gene for the synthesis of the active form jasmonoyl-isoleucine (Staswick and Tiryaki, 2004). As noted above, there was also a JA pathway activated by NO independently of RAP2.3 that involved the function of a biosynthetic module comprised of LOX3, AOC1 or AOC3, and OPR3, and a signaling module comprising the activator MYC2 and the repressors JAZ5, 6, 7, and 10 (Fig. 5, Supplementary Table S2). Notably, despite in principle being regulated by NO independently of RAP2.3, the biosynthetic *AOC1* gene and the regulatory *MYC2* and *JAZ6* genes contained (C/A)GCCG(C/T)(C/A) motifs in their promoters (Supplementary Table S3). Similarly, genes related to ABA biosynthesis and signaling were also included in this set. The biosynthetic *BCH1* and *ABA1* genes coding for β-carotene hydroxylase 1 and zeaxanthin epoxidase, respectively, were down-regulated by NO and the metabolic *UGT71B6* gene coding for UDP-glucosyl transferase 71b6 was up-regulated by NO only in TPT_RAP2.3 plants that were not treated with EST (Fig. 6). Only the promoter of *UGT71B6* contained the (C/A)GCCG(C/T)(C/A) motif (Supplementary Table S3). We also found that only some genes coding for regulatory components of the core ABA signaling were regulated by NO, and some of them were dependent and other independent of *RAP2.3* expression (Supplementary Table S2). The ABA genes encoding the receptors PYL7 and PYL4 were up-regulated by NO and this induction was repressed by RAP2.3 (Fig. 6, Supplementary Table S2). The gene coding for the PYL5 receptor was strictly dependent on RAP2.3 overexpression for NO-induced expression, and the gene encoding PYL6 was up-regulated by NO independently of RAP2.3. Taken together, these results suggest that NO may control ABA perception through gene-specific pathways with or without regulation by RAP2.3. This type of gene-specific regulatory effect could also be applied to the positive protein kinase regulators encoded by the *SnRK2* gene family. Only *SnRK2.3* and *SnRK2.9* were up-regulated by NO, with the induced expression of *SnRK2.3* being abolished by overexpression of RAP2.3 whereas *SnRK2.9* expression was not modulated by RAP2.3 (Fig. 6, Supplementary Table S2). Only the promoters of genes coding for the positive ABA regulators PYL7, SnRK2.3, and SnRK2.9 contained the (C/A)GCCG(C/T)(C/A) motif (Supplementary Table S3). However, the existence of gene-specific branch pathways inside the ABA signaling process did not match with the expression patterns of ABA target genes coding for either signaling components or transcription factors, which were mostly up-regulated by NO through a RAP2.3 repression mechanism (Fig. 6). Only *SLAH2* and *AIB*, coding for a nitrate-specific anion channel and an ABA-inducible bHLH-type transcription factor, respectively, were up-regulated by NO independently of RAP2.3 (Fig. 6, Supplementary Table S2). Moreover, we found (C/A)GCCG(C/T)(C/A) motifs not only in the promoters of genes up-regulated by NO only in the absence of *RAP2.3* overexpression, such as *MYB51* and *LTI30*, but also in *SLAH2*, which was up-regulated by NO independently of *RAP2.3* expression levels. Our findings therefore suggested that RAP2.3 exerted a very efficient repression of the NO-induced expression of ABA target genes either through the direct regulation of key targets by binding the promoters containing (C/A)GCCG(C/T)(C/A) motifs, or alternatively by regulating just a master ABA signaling component, probably coding for a transcription factor. To determine whether JA- and ABA-related genes regulated by RAP2.3 were associated with phenotypic changes in the sensitivity to these hormones, we examined primary root elongation in the presence or absence of each. No significant changes were detected in the sensitivity to JA-induced root shortening in any of the plants with gain of function of RAP2.3, or in single or multiple *erfvii* mutants (Supplementary Fig. S4). In turn, the double *rap2.3 rap2.12* and quintuple *qerfvii* mutants together with the *MA-RAP2.3*-OX plants overexpressing the undegradable version of RAP2.3 were all hypersensitive to ABA, whereas the *rap2.3* single-mutant and *MC-RAP2.3* wild-type responded to ABA in the same way as the wild-type (Supplementary Fig. S4). These results suggested a complex pattern of regulation of ABA signaling by NO and ERFVIIs, probably with *ERFVII* gene-specific effects on this phenotype.

### NO-regulated expression of JA- and ABA-related genes is increased by loss of ERFVII function and reduced by gain of ERFVII function

**Fig. 7.**
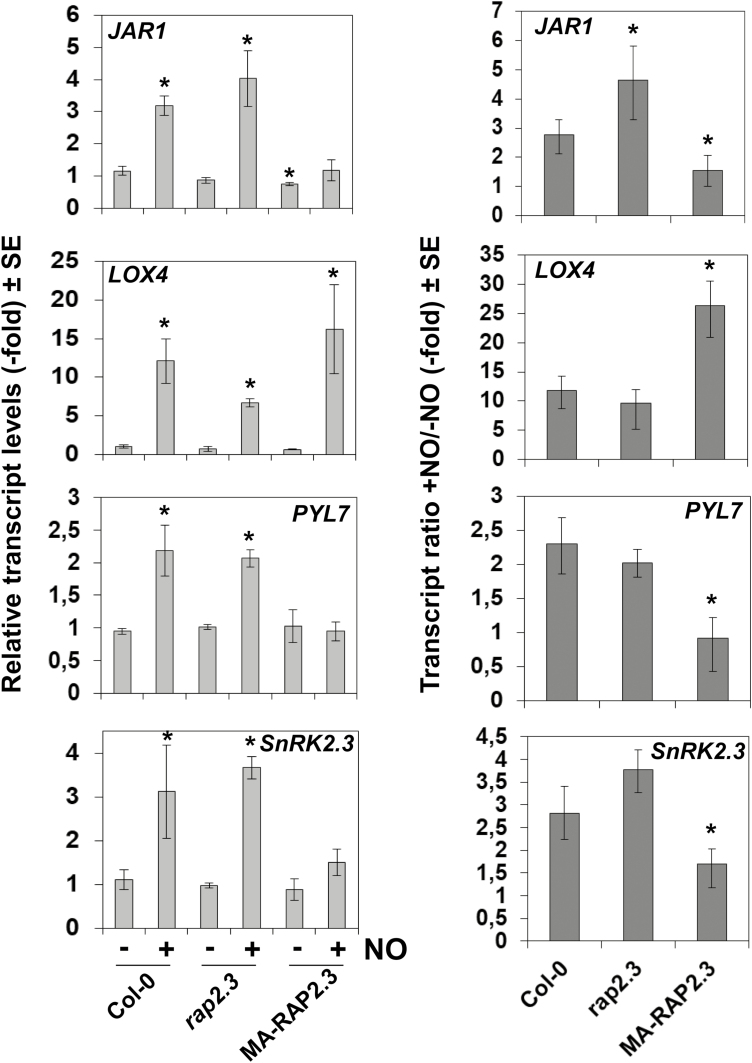
NO-triggered transcript induction of genes related to jasmonates and ABA in Arabidopsis plants with loss or gain of RAP2.3 function. Levels of transcripts were quantified by RT-qPCR in the Col-0 wild-type, the *rap2.3* mutant (loss of function) and transgenic *MA-RAP2.3*-overexpression plants (producing a non-degradable form of the protein) either with (+) or without (–) treatment with NO. The *Actin 2* (*ACT2*) gene was used to normalize the results. The transcript levels are expressed relative to the value in Col-0 without NO, which was set as 1 (left), and the ratio of transcripts between the +NO and –NO treatments is also shown (right). Data are means of three independent replicates. Significant differences compared with Col-0(–NO) or Col-0 were determined using Student’s *t*-test: * *P*<0.05.

To validate the regulation that RAP2.3 exerted on some of the JA- and ABA-related genes identified in the transcriptome analyses, we conducted RT-qPCR analysis of the corresponding transcripts in NO-treated wild-type and *qerfvii* mutant plants. We found that NO-triggered up-regulation of *JAR1*, *PYL7*, and *SnRK2.3* was increased in *qerfvii* compared to the wild-type (Supplementary Fig. S5), which was in agreement with ERFVIIs reducing the up-regulation of these genes by NO (Figs 5, 6, Supplementary Table S2). In turn, the up-regulation of *JAZ6* and *LOX4* by NO was reduced in *qerfvii* plants (Supplementary Fig. S5), which was also consistent with the enhanced up-regulation detected in TPT_RAP2.3 plants treated with EST (Fig. 5 and Supplementary Table S2). Also consistent with the transcriptome analysis, the up-regulated expression of *LOX3*, *JAZ10*, *PYL6*, and *SnRK2.9* by NO was not altered in *qerfvii* plants (Supplementary Fig. S5). Among the genes examined by qPCR only *PYL5* showed a different pattern to that detected in the transcriptome analysis, with similar transcript levels in wild-type and *qerfvii* plants, with its up-regulation in response to NO mainly being independent of RAP2.3. We also confirmed that only a subset of genes carrying RAP2.3 binding sites in their promoters seemed to be directly regulated by RAP2.3, and these included *JAR1*, *LOX4*, *PYL7*, and *SnRK2.3*,. Consistent with the results from the transcriptome analysis, *JAR1*, *PYL7*, and *SnRK2.3* displayed defective NO-triggered induction in *MA-RAP2.3*-OX plants (Fig. 7), whilst induction of *LOX4* was increased. In *rap2.3* mutant plants only *JAR1* had significantly higher induction in response to NO.

**Fig. 8.**
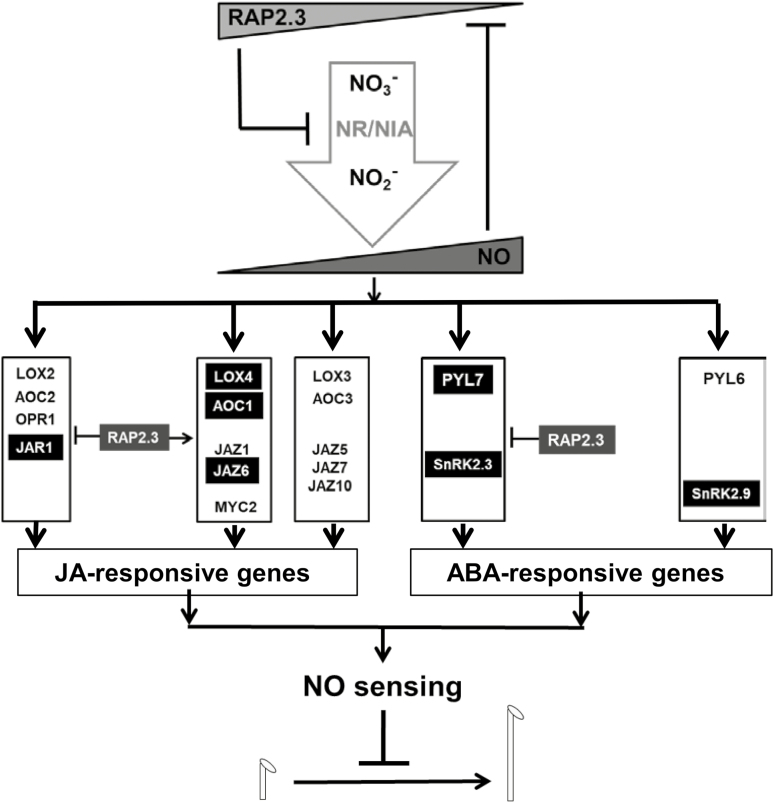
A proposed model for the regulation of NO-triggered jasmonate (JA) and ABA synthesis and signaling pathways through a RAP2.3-based rheostat-like mechanism that controls nitrate reductase (NR/NIA)-mediated NO biosynthesis and NO-regulated hypocotyl growth. Potential direct targets of RAP2.3 containing the putative RAP2.3-binding site in their gene promoters are highlighted in black. An arrow indicates promotion of a pathway by RAP2.3 whilst a blocked line indicates repression.

Overall, our results suggest the existence of two NO-responsive branch pathways of JA and ABA signaling, one of which is RAP2.3-dependent and the other RAP2.3-independent (Fig. 8). Three different branches of JA signaling can be proposed based on the regulatory role exerted by RAP2.3. A first RAP2.3-repressed branch would involve LOX2, AOC2, OPR1, and JAR1, of which only JAR1 would be a direct RAP2.3 target. A second RAP2.3-activated JA signaling branch would recruit LOX4, AOC1, JAZ1, JAZ6, and MYC2, of which LOX4, AOC1, and JAZ6 would be potential direct targets of RAP2.3. Finally, a third RAP2.3-independent branch would involve the participation of LOX3, AOC3, JAZ5, JAZ7, and JAZ10. The NO-sensitive ABA signaling pathway would be split into two branch pathways (Fig. 8). The first, repressed by RAP2.3, would involve PYL7 and SnRK2.3, both having potential RAP2.3 binding sites in their promoters, and the second branch would comprise the function of the PYL6 receptor and SnRK2.9 kinase acting through a RAP2.3-independent mechanism.

## Discussion

NO is an important regulator of plant responses to stress (Arasimowicz-Jelonek and Floryszak-Wieczorek, 2014; Fancy *et al.*, 2017) and is also crucial in controlling many developmental transitions (He *et al.*, 2004; Lozano-Juste and León, 2011; Arc *et al.*, 2013). We have previously demonstrated a regulatory role for NO in regulating responses related to abiotic stress, mainly through actions on ABA perception and signaling (Lozano-Juste and León, 2010; León *et al.*, 2014; Castillo *et al.*, 2015), but also through its antagonism of gibberellin signaling through stabilization of DELLA proteins (Lozano-Juste and León, 2011). Furthermore, we have also demonstrated that NO is required for cysteine 2 oxidation-dependent polyubiquitylation and subsequent proteasome-mediated degradation of RAP2.3, a process that forms the basis of a NO-sensing mechanism in Arabidopsis (Gibbs *et al.*, 2014*b*). This mechanism can also integrate environmental factors such as soil salinity to aid plant survival via ERFVII-mediated processes that involve interactions with chromatin-remodeling events (Vicente *et al.*, 2017). Despite all the information accumulated to date, the functional interaction between NO and RAP2.3 in controlling plant responses at the transcriptome level has remained unclear. In this study, our findings indicate that there is a regulatory loop involving NO and RAP2.3 that controls how plants respond to NO. We found that RAP2.3 acts mostly as a repressor of NO-triggered responses both at the physiological (Figs 1, 3) and molecular (Fig. 4D, Supplementary Table S2) levels.

Transcription factors of the AP2-ERF multigene family can be classified in 12 groups in Arabidopsis (Nakano *et al.*, 2006). Some of those in the groups II, III, and VIII contain the Ethylene response factor-associated Amphiphilic Repression (EAR) motif (Ohta *et al.*, 2001), which has been reported to act in transcriptional repression via recruitment of chromatin remodeling factors that facilitate epigenetic regulation of gene expression (Kagale and Rozwadowski, 2010, 2011). Some of the AP2/ERF transcription factors containing EAR motifs, such as DEAR1 and RAP2.1, have been reported to act as transcriptional repressors to control stress responses (Tsutsui *et al.*, 2009; Dong and Liu, 2010). RAP2.3, however, does not contain an EAR motif, so its potential activity as a repressor in NO-triggered responses would be expected to be indirect via its activation of a true repressor. But we found that none of the genes that were up-regulated by NO in TPT-RAP2.3 plants only after β-estradiol (EST) treatment and that contained the putative RAP2.3-binding motif (Supplementary Table S3) coded for an EAR-containing transcription factor. As an alternative hypothesis, the attenuated NO-triggered response at the transcriptome level could have been the result of a NO-induced transcriptional activator that was down-regulated upon overexpression of *RAP2.3*. Among the candidates of this type, three genes coding for AP2/ERF transcription factors (*ERF095/ESE1*, *RAP2.6*, and *ERF016*) were strongly activated by NO only when *RAP2.3* was not overexpressed (Supplementary Table S2). It has recently been reported that RAP2.6 is activated in Arabidopsis after plants are treated with the NO donor S-nitrosocysteine, and also that *rap2.6* mutant seedlings are partially insensitive to NO in inhibiting shoot elongation (Imran *et al.*, 2018), which would fulfill the features required of a RAP2.3-repressed target responsible for NO-triggered responses. Moreover, the RAP2.6 promoter contained a putative RAP2.3-binding motif (Supplementary Table S3).

In addition to AP2/ERFs, NAC055/NAC3 and MYB113 were also up-regulated by NO only when *RAP2.3* was not overexpressed and they also contained the putative RAP2.3-binding domain in their promoters (Supplementary Tables S2, S3). NAC055 has been reported to participate in JA signaling downstream of MYC2 (Bu *et al.*, 2008). We found that NO induced the expression of a subset of JA-related genes coding for its biosynthesis and signaling through a process that was repressed by RAP2.3 in a gene-specific manner (Fig. 6). The existence of a NO-induced RAP2.3-repressed JA biosynthesis pathway (Fig. 8) could be determined by the regulatory function exerted by NAC055. This pathway would involve *JAR1*, which contained a RAP2.3 binding site in its promoter (Supplementary Table S3); the gene was up-regulated by NO, and this up-regulation was increased in *rap2.3* and *qerfvii* plants (Fig. 7, Supplementary Fig. S5) and reduced in plants overexpressing the non-degradable MA-RAP2.3 protein (Fig.7) and in TPT_RAP2,3 plants treated with EST (Supplementary Table S2). It is known that *JAR1* plays a crucial role in the biosynthesis of the active hormone jasmonoyl-isoleucine (Staswick *et al.*, 2002). It has recently been reported that an antagonistic relationship between JAR1/FIN219 and CRY1 modulates photomorphogenesis under blue-light conditions (Chen *et al.*, 2018), a process that we have previously demonstrated also to be regulated by endogenous NO (Lozano-Juste and León, 2011). However, we did not identify any significant changes in JA sensitivity in root elongation assays for plants either not expressing or overexpressing *RAP2.3* or other *ERFVIIs* (Supplementary Fig. S4), suggesting that the potential effects are more relevant in shoots than in roots. Similar to JA signaling, a branch of the ABA signaling pathway also seemed to be sensitive to NO and repressed by RAP2.3 (Fig. 8). This branch would involve PYL7 and SnRK2.3, the genes of both of which contain putative RAP2.3 binding sites in their promoters (Supplementary Table S3), and both of which lacked up-regulation in plants overexpressing non-degradable RAP2.3 (Fig. 7) and displayed greater up-regulation in the *qerfvii* mutant (Supplementary Fig. S5). ABA signaling seems to be modulated through the control of SnRK2.3 proteasomal degradation mediated by SCFAtPP2b-11 (Cheng *et al.*, 2017). *PP2b11* and its homolog *PP2b-13* were up-regulated by NO only when RAP2.3 is not overexpressed (Supplementary Table S2), suggesting that this process may represent a RAP2.3-mediated mechanism to control both the expression and the stability of SnRK2.3 in response to stress factors that produce NO. Moreover, NO has been previously reported to repress the function of SnRK2.3 by a post-translational modification based on S-nitrosylation of cysteine residues (Wang *et al.*, 2015). Some of these ABA-responsive genes were also found to be regulated by other stress-related hormones such as JA and SA, thus pointing to NO as an enhancer of general RAP2.3-independent stress responses in the plant (Zhang *et al.*, 2006; Singh *et al.*, 2017). Some of the genes considered above contained (C/A)GCCG(C/T)(C/A) motifs in their promoter sequences (Supplementary Table S3), suggesting not all the motifs identified *in silico* were truly RAP2.3-related, and/or that RAP2.3 binding on these motifs did not modify the regulation exerted on them by NO. Moreover, we detected significant hypersensitivity to ABA in the roots of plants mutated either in several or all ERFVIIs but not in the roots of the *rap2.3* single-mutant, and we also found hypersensitivity in plants that overexpressed the MA-RAP2.3 version that is not degraded through the PRT6 N-degron pathway (Supplementary Fig. S4). These findings point to a complex regulation exerted on ABA signaling by ERFVIIs, with combinatorial actions resulting in gene-specific phenotypic alterations in different plant organs and/or physiological conditions.

NO modulated its biosynthesis and triggered responses including JA and ABA signaling through both RAP2.3-independent and RAP2.3-dependent pathways. A remarkable feature of the RAP2.3 regulatory function on NO signaling was that it mostly acted as a repressor of gene-specific branches of several signaling pathways. It is worth mentioning that this type of regulation was not exclusive to RAP2.3, as we detected similar repressive roles on gene expression by another member of the ERFVII group, RAP2.12 (Supplementary Fig. S2). Interestingly, the way RAP2.3 exerted regulatory effects on NO-triggered responses was partially based on its capacity to be degraded by the proteasome. Conditions that blocked its degradation, such as in a *prt6* mutant background or the overexpression of the non-degradable MA-RAP2.3 version, reduced the sensitivity of the plants to NO (Fig. 3), thus suggesting that NO signaling would be modulated by using RAP2.3 as a form of rheostat. Biological rheostats have been defined as biological process that allow the control of a signaling mechanism or physiological response in a graduated quantitative manner in opposition to the on–off binary function of a switch (Mrosovsky, 1990). The rheostat concept has recently been proposed to define the function of ABA receptor–PP2C phosphatase pairs to integrate the fluctuating ABA levels under stress conditions (Tischer *et al.*, 2017). Several other key processes in plant biology are controlled through rheostat-like mechanisms. ROS homeostasis is subjected to a negative-feedback control through the so-called ROP–GAP rheostat, which determines the adaptation of plants to low oxygen availability (Baxter-Burrell *et al.*, 2002). Phytoene desaturase and DNA demethylase have also been described as rheostats in Arabidopsis in the control of the retrograde signaling in chloroplast biogenesis and in epigenetic regulation, respectively (Foudree *et al.*, 2010; Williams *et al.*, 2015). Our findings suggest that RAP2.3 plays not only its previously proposed role in sensing NO, but also functions as a NO- and O_2_-modulated rheostat (Fig. 8) that integrates environment-triggered changes in the endogenous levels of NO and oxygen-containing molecules, as well as some hormones such as jasmonates and ABA.

## Supplementary data

Supplementary data are available at *JXB* online.

Fig. S1. NO in roots of plants overexpressing MC-RAP2.3 and MA-RAP2.3, and in mutants related to the N-degron pathway.

Fig. S2. Comparison of DEGs in response to NO in plants conditionally expressing RAP2.12 or RAP2.3.

Fig. S3. Sensitivity to cytokinin and auxin in primary root elongation assays for ERFVII-overexpressing and mutant plants.

Fig. S4. Sensitivity to ABA and JA in primary root elongation assays for ERFVII-overexpressing and mutant plants.

Fig. S5. NO-triggered transcript induction in wild-type and *qerfvii* mutant plants.

Table S1. Oligonucleotides used in this work.

Table S2. Differential transcript levels in NO-treated TPT_RAP2.3 and TPT_RAP2.12 lines with or without induction by β-estradiol.

Table S3. *In silico* genome-wide analysis of putative RAP2.3-binding motifs and the intersection with NO-regulated genes.

eraa069_suppl_supplementary_figures_S1_S5_table_S1

eraa069_suppl_supplementary_table_S2

eraa069_suppl_supplementary_table_S3
