# Supplementary material for: RAP2.3 negatively regulates nitric oxide biosynthesis and related responses through a rheostat-like mechanism in Arabidopsis
Source: J Exp Bot. 2020 Feb 13;71(10):3157–71. doi: 10.1093/jxb/eraa069 (PMC7260729; doi:10.1093/jxb/eraa069)
Supplement: eraa069_suppl_supplementary_figures_S1_S5_table_S1 [file eraa069_suppl_supplementary_figures_s1_s5_table_s1.pdf]

Title: RAP2.3 negatively regulates nitric oxide biosynthesis and related responses through a rheostat-like mechanism in Arabidopsis

Short title: RAP2.3 represses NO action

Authors: José León, Álvaro Costa-Broseta, Mari Cruz Castillo

Supplementary data

Figure S1. NO in roots of (A) plants overexpressing MC- and MA-RAP2.3 versions, and (B) N-end rule pathway-related mutants.

Figure S2. Comparison of DEGs in response to NO in plants conditionally expressing RAP2.12 or RAP2.3.

Figure S3. Sensitivity to cytokinin and auxin in primary root elongation assays with mutant and overexpressing ERFVII plants.

Figure S4. Sensitivity to ABA and JA in primary root elongation assays with mutant and overexpressing ERFVII plants.

Figure S5. NO-triggered transcript induction in wild type and *qerfvii* mutant plants.

Table S1. Oligonucleotides used in this work.

Figure S1

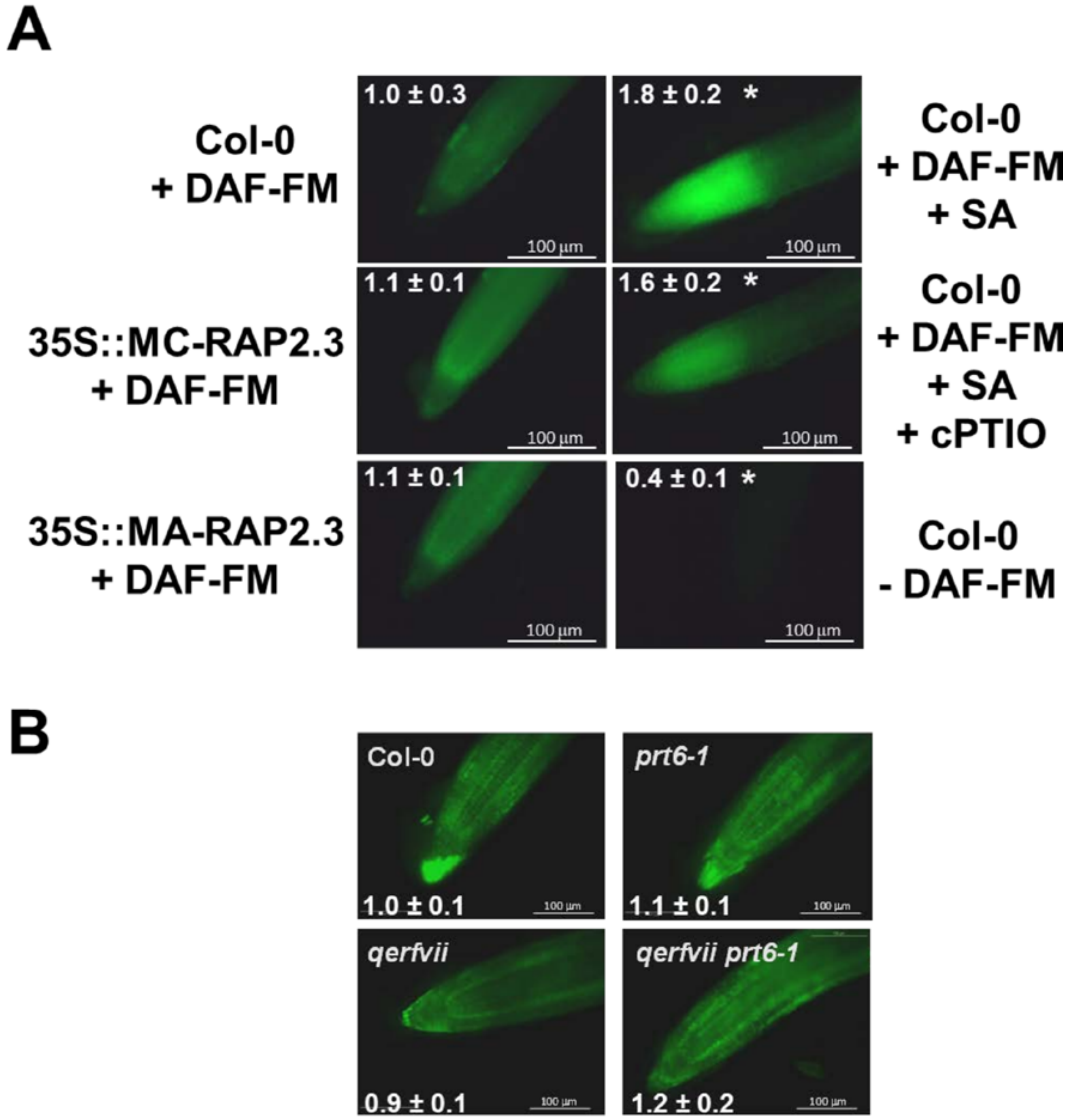

Supplementary Fig. S1 NO in roots of (A) plants overexpressing MC- and MA-RAP2.3 versions, and (B) N-end rule pathway-related mutants. Roots from plants of the indicated genotypes were treated (+) or untreated (-) with 10 μM DAF-FM DA, 250 μM of the NO scavenger cPTIO, and 100 μM of the NO inducer salicylic acid (SA) as indicated. Fluorescence was detected by fluorsecence microscopy with identical setup for all the genotypes and conditions tested. Images are representative of three to five replicate experiments.

Supplementary Figure S2

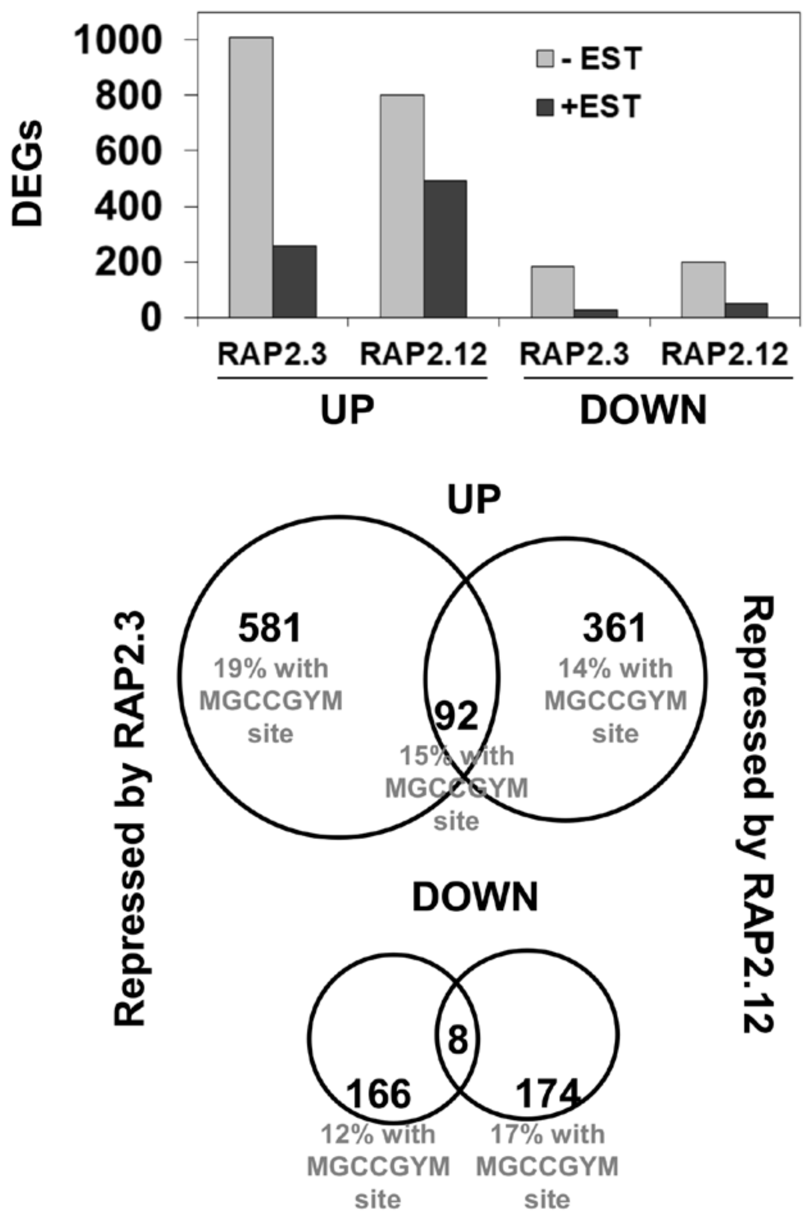

Supplementary Figure S2. Comparison of DEGs in response to NO in plants conditionally expressing RAP2.12 or RAP2.3. The transcriptome of NO-treated TPT_RAP2.3 and TPT_RAP2.12 plants were analyzed upon β-estradiol induced (+EST) or not induced (-EST) expression of transgenes. Overlapping between gene subsets was analyzed by using Venny 2.0, and the presence of MGCCGYM motifs in the promoter was analyzed by using the Patmatch tool from TAIR.

Supplementary Figure S3

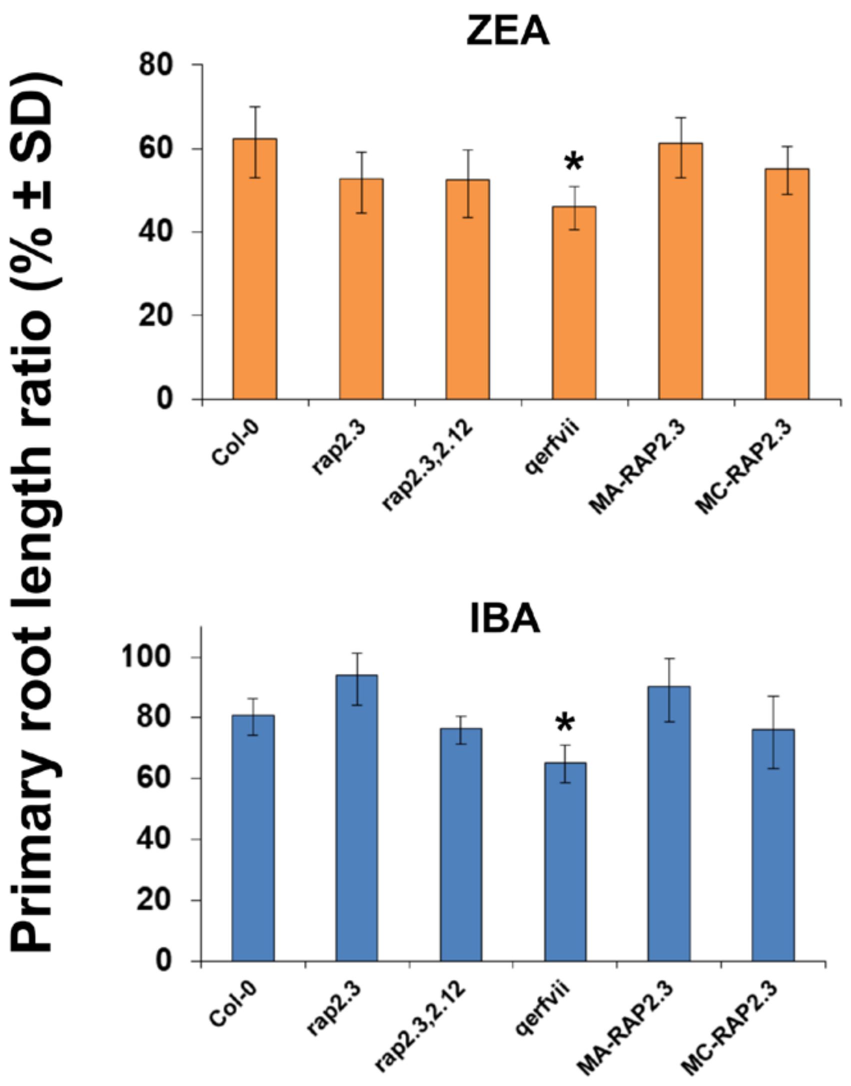

Supplementary Figure S3. Sensitivity to cytokinin and auxin in primary root elongation assays with mutant and overexpressing ERFVII plants. Seed lings of the indicated geneotypes were germinated and grown for 5 days in vertical MS plates. Then, 15 seedlings per genotype were transferred to either MS or MS supplemented with 10 uM zeatin (ZEA) or indolebutyric acid (IBA) and grown in vertical plates for additional 5 days. After scanning the length of the primary roots during treatment was quantified by using ImageJ. Values are the percentage ratio of the root length in treated versus untreated seedlings ± standard deviation. * represents statistically significant with p value < 0.05 in Student´s t-test.

Supplementary Figure S4

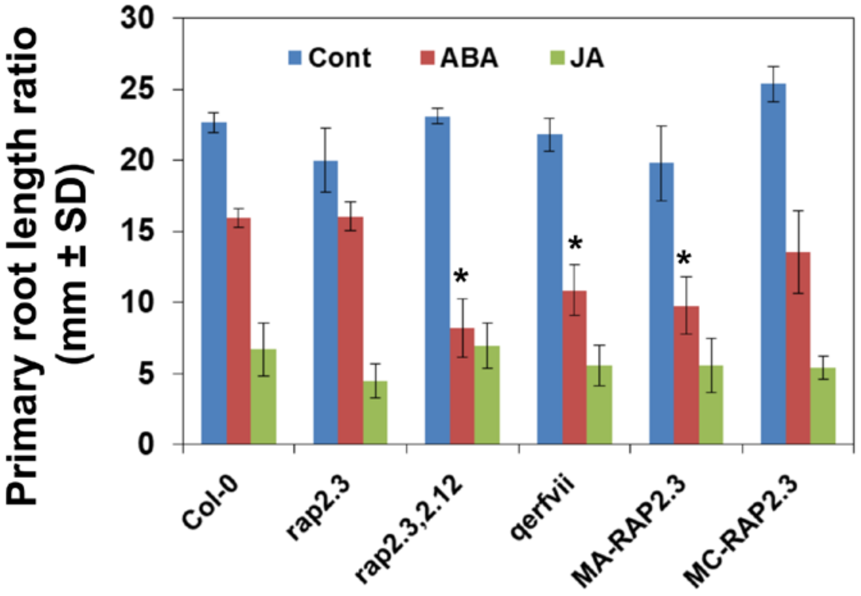

Supplementary Figure S4. Sensitivity to ABA and JA in primary root elongation assays with mutant and overexpressing ERFVII plants. Seed lings of the indicated geneotypes were germinated and grown for 5 days in vertical MS plates. Then, 15 seedlings per genotype were transferred to either MS (Cont) or MS supplemented with 10 uM abscisic acid (ABA) or jasmonic acid (JA) and grown in vertical plates for additional 5 days. After scanning the length of the primary roots during treatment was quantified by using ImageJ. Values of the root length are expressed in mm ± standard deviation. * represents statistically significant compared to Cont untreated seedlings with p value < 0.05 in Student´s t-test.

Supplementary Figure S5

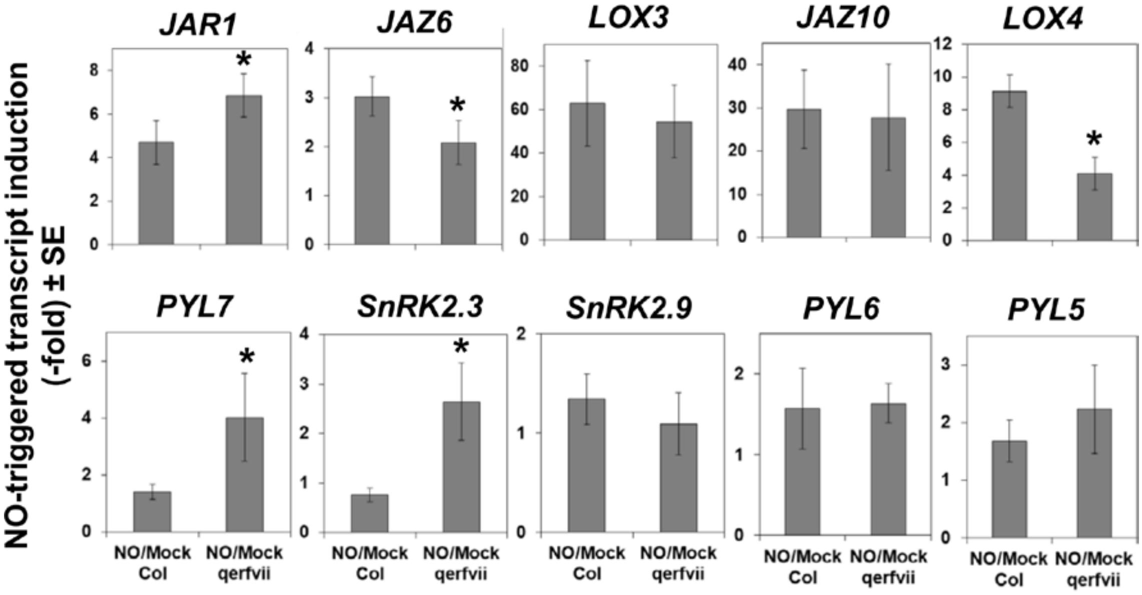

Supplementary Figure S5. NO-triggered transcript induction in wild type and *qerfvii* mutant plants. Levels of transcripts were quantified by RT-qPCR with specific primers described in Supplementary Table S1. Values are the mean of three independent replicates ± standard error. * represents statistically significant with p value < 0.05 in Student´s t-test.

**Supplementary Table S1.** Oligonucleotides used in this work.			
**Name**	**Sequence (5´to 3´)**	**AGI**	**Application**
qP-ACT2-R	TGTCTCGTGGATTCCAGCAG	AT3G18780	qRT-PCR
qP-ACT2-F	TTGTTCCAGCCCTCGTTTGT	AT3G18780	qRT-PCR
qRAP2.3-R	TCTGTTGCCTGCTCCTTCTTCACT	AT3G16770	qRT-PCR
qRAP2.3-F	CAAACTCCATCCCACCAACCAAGT	AT3G16770	qRT-PCR
qRAP2.12-R	GCTGCGGAAGGTTCAGTTTTTGGT	AT1G53910	qRT-PCR
qRAP2.12-F	TGCAGATTTCTCAGCGTCCCCATC	AT1G53910	qRT-PCR
qPYL5-F	GGTCACCGGTGCAACTCC	At5g05440	*qRT-PCR*
qPYL5-R	CGCGTGGATCATCTGCACC	At5g05440	qRT-PCR
qPYL6-F	CCAACGTCGATACAGTTTCAG	At2g40330	qRT-PCR
qPYL6-R	CCTCCACGTCTTGTACCACG	At2g40330	qRT-PCR
qPYL7-F	GATCGGAGGAGACGATACAGATACA	At4g01026	qRT-PCR
qPYL7-R	AGTGGTGAAGATGACGCAACCT	At4g01026	qRT-PCR
qSnRK2.3-F	TCGAATTTCTCTTTTTGTGATCAGA	At5g66880	qRT-PCR
qSnRK2.3-R	ACTGTCGTGCATAATCGGCATA	At5g66880	qRT-PCR
qSnRK2.9-F	GGAGAAGTATGAGATGGTGAAGGATT	At2g23030	qRT-PCR
qSnRK2.9-R	CACAAGCTCGTTTGTTTGCTTATT	At2g23030	qRT-PCR
qLOX3-F	CGGATAGAGAAAGAGATTGAGAAAAGGAAC	At1g17420	qRT-PCR
qLOX3-R	AGGTACACCTCTACACGTAACACCAGGC	At1g17420	qRT-PCR
qLOX4-F	CCCTTTTGATCCGAGACCTATG	At1g72520	qRT-PCR
qLOX4-R	CTTCACCGACTTCTCCTCTTCTTC	At1g72520	qRT-PCR
qJAR1-F	GCCAAGATGTGTGAAGCCAAG	At2g46370	qRT-PCR
qJAR1-R	AAAACGCTGTGCTGAAGTAGC	At2g46370	qRT-PCR
qJAZ6-F	TCATCTTCCTCCCAAGCCAGAGAT	At1g72450	qRT-PCR
qJAZ6-R	ACTAGAAACGTGAACTCGATCGTGCAT	At1g72450	qRT-PCR
qJAZ10-F	TCGCAAGGAGAAAGTCACTGCAAC	At5g13220	qRT-PCR
qJAZ10-R	CGATTTAGCAACGACGAAGAAGGC	At5g13220	qRT-PCR
